# Management of Hyperlipidaemia using KNEA: A case report

**DOI:** 10.6026/973206300212697

**Published:** 2025-08-31

**Authors:** Nikil Niva J., Hema Nandhini Devi V., Sridevi L., Susila R., Muthukumar N.J.

**Affiliations:** 1 Department of Outpatient, Siddha Clinical Research Unit, Central Council for Research in Siddha, Safdarjung Hospital, New Delhi, India; 2 Pharmacovigilance, Peripheral Pharmacovigilance Centre, Siddha Clinical Research Unit, Central Council for Research in Siddha, Safdarjung Hospital, New Delhi, India; 3 Outpatient Department, Siddha Clinical Research Unit, Central Council for Research in Siddha, Safdarjung Hospital, New Delhi, India; 4 Siddha Clinical Research Unit, Central Council for Research in Siddha, Safdarjung Hospital, New Delhi, India; 5 Director General, Central Council for Research in Siddha, Ministry of Ayush, Govt of India, Tambaram Sanatorium, Chennai, India

**Keywords:** Case report, hyperlipidaemia, siddha medicine, ayush

## Abstract

Elevated lipid levels in the blood, often termed Hyperlipidaemia, significantly raise the risk of developing atherosclerotic cardiovascular disease (ASCVD). Dyslipidemia is widely recognized as a key factor in the development of cardiovascular diseases and stroke. The present case report shows a patient with hyperlipidemia who was successfully treated with Siddha therapeutic regimens KNEA (*Keelanelli Maathirai*, *Nerunjil Kudineer*, *Eladhi Chooranam Maathirai*, *Ayabirungaraja Karpam*). The therapeutic medications have its own Antihyperlipidemic, hepatoprotective, antioxidant and nephroprotective properties. In this case report, total cholesterol (TC) was reduced by 178 mg/dL, dropping from 205 mg/dL, while low-density lipoprotein cholesterol (LDL-C) decreased by 108 mg/dL, going down from 127 mg/dL. Additionally, the HDL-C level increased from 33 mg/dL to 42 mg/dL. Thus, the use of KNEA in the management of Hyperlipidaemia through Siddha medications is shown.

## Background:

Hyperlipidaemia is characterized by an imbalance in blood cholesterol levels, specifically an elevated in low-density lipoprotein (LDL) cholesterol and a reduction in high-density lipoprotein (HDL) cholesterol [1]. There are several types of hyperlipidaemia, including pure hypercholesterolemia, which involves high cholesterol levels alone; mixed hyperlipidaemia, marked by elevated levels of triglycerides and cholesterol simultaneously; and hypertriglyceridemia, which refers specifically to high triglyceride levels [1]. Elevated lipid levels in the blood, often termed hyperlipidaemia, crucially raise the risk of developing atherosclerotic cardiovascular disease (ASCVD) [1]. This condition can lead to severe cardiovascular events, such as coronary artery disease (CAD), myocardial infarction (MI), strokes, and diseases affecting the arteries in the limbs [1]. Dyslipidemia is widely recognized as a crucial element in the progress of CVD and stroke. Multiple factors can elevate the risk of hyperlipidemia [2]. Obesity, physical inactivity, smoking habits, and diets high in saturated or trans-fats are considered modifiable risk factors [3]. Additionally, chronic kidney disease, systemic hypertension, type 2 diabetes mellitus, hypothyroidism, and obstruction of the biliary tracts are recognized as secondary causes of increased levels of LDL cholesterol [3]. When total cholesterol levels are elevated to 240 mg/dl or triglyceride levels rise to 200 mg/dl, the risk of atherosclerotic events increases [4]. In India, the prevalence of hypercholesterolemia varies across different studies, indicating rates of 10 to 15% in rural areas and 25-30% in urban regions [5].

The ICMR-INDIAB also identified that hypertriglyceridemia is notably more common in both gender in urban populations compared to rural ones, with average levels recorded at 149 ± 2.05 vs. 136 ± 1.07 mg/dl, and hypertriglyceridemia rates at 36.4% vs. 30.0% [5]. This prevalence is significantly higher than that of hypercholesterolemia [5]. The main treatments for hyperlipidemia are statins and ezetimibe, while bile acid sequestrants (BAS) are used less often. PCSK9 inhibitors can be utilized alone or alongside statins for optimal lipid control [1]. Traditional herbal medicine (THM) represents a significant component of alternative and complementary medicine for managing metabolic disorders [6]. The Siddha System of Medicine is a unique traditional medical system in India, with its origins dating back to before 4000 BCE [7 ]. Numerous plants in Siddha medicine have demonstrated significant benefits in managing hyperlipidemia, a condition categorized by elevated lipid levels in the blood. Various Siddha formulations have been shown to effectively reduce these lipid levels, often without causing serious side effects [8]. Therefore, it is of interest to report a patient with Hyperlipidaemia who was successfully treated with Siddha therapeutic regimens KNEA.

## Case presentation:

## Patient Information:

On October 24, 2024, a 38-year-old male clinical laboratory worker visited the Outpatient Department (OPD) at the Siddha Clinical Research Unit in Safdarjung Hospital, New Delhi (SCRUND). He reported experiencing a mild burning sensation in the epigastric and right hypochondriac regions, along with occasional abdominal pain over the past two months. The patient had no history of diabetes, thyroid dysfunction, systemic hypertension, or any other comorbidities or autoimmune disorders. He also reported no history of smoking, alcohol consumption, or pan chewing, and there was no relevant family or psychosocial history of diseases. The patient followed a vegetarian diet. During a regular check-up in December 2023, an ultrasound of the abdomen and pelvis revealed that the patient had Grade II fatty liver, and his lipid profile indicated abnormalities. He adhered to the statin medications prescribed by his allopathic physician; however, his lipid levels did not return to normal, and other clinical features persisted. In a follow-up appointment on October 23, 2024, laboratory blood tests showed elevated lipid levels. Consequently, the patient returned to the outpatient department (OPD) at SCRUND, New Delhi, for further management.

## Clinical findings:

At the time of the patient's visit, he reported experiencing a burning sensation in the epigastric and right hypochondriac regions. During the general examination, the patient's sleep, appetite, bladder, and bowel movements were all normal. The pulse rate- 79 beats per minute, BP: 130/80 mm Hg, and body temperature was within the normal range. Height was 176 cm; weight was 70 kg and BMI was 22.6 kg/m^2^. Upon systemic examination, the cardiorespiratory, musculoskeletal, neurological, and genitourinary systems appeared normal. In the abdominal examination, mild tenderness was observed upon examination in the right lumbar and right upper abdominal regions. Normal movements were observed during respiration, and no dull sounds were detected during percussion. Additionally, bowel sounds were found to be normal upon auscultation. According to Siddha medicine, the patient was examined using the traditional eight-fold diagnostic method. The assessments conducted were as follows: tongue examination- normal, examination of the color of the body - normal, examination of skin -normal, tongue examination- normal, no abnormal sound detected, eye examination - normal, stool examination - normal, urine examination- normal. As per Siddha medicine examination methods Overall, the findings from all examinations were normal.

## Timeline:

Table 1 (see PDF) presents the timeline of events in the current case study, including symptoms, previous patient treatment, and Siddha interventions.

## Diagnostic assessment:

On October 23, 2024, the blood lipid profile showed the following values: Total Cholesterol (TC) was 205 mg/dL, Triglycerides (TGs) were 301 mg/dL, Low-Density Lipoprotein Cholesterol (LDL-C) was 127 mg/dL, and High-Density Lipoprotein Cholesterol (HDL-C) was 33 mg/dL. The lipid markers indicated moderate deviations from normal levels [9]. The patient has a previous history of hyperlipidemia and was diagnosed with grade II fatty liver a year prior. Considering the blood investigations, medical history, and clinical features, the patient was diagnosed with moderate hyperlipidemia.

## Therapeutic intervention:

In preparation for Siddha treatment, all allopathic medications were discontinued. The following three Siddha sastric medicines were prescribed to the patient. 1. *Keelanelli Maathirai* (KM) (medicated herbal tablet- 500mg) 2 tablets twice a day (morning and night) before food with warm water. *Eladhi Chooranam Maathirai* (ECM) (medicated herbal tablet- 500mg) 2 tablets twice a day (morning and night) after food with warm water. *Ayabirungaraja Karpam* (ABK) (medicated rejuvenating drug) 500 mg twice a day (morning and night) after food with honey. 30 ml of *Nerunjil Kudineer* (NMK) (medicated herbal decoction) twice a day (morning and night) after food.

## Follow-up and outcomes:

The patient was directed to attend the hospital once every seven days and was encouraged to follow a regular diet. During each visit, assessments of the patient's condition were documented. Throughout the Siddha treatment period, there were no reported adverse drug reactions, and the patient completed the medication without any complications. After two weeks of treatment, the patient was advised to undergo a lipid profile blood test. On November 6, 2024, the blood investigations revealed the following lipid levels: Total Cholesterol (TC) was 190 mg/dL, TGs were 163 mg/dL, LDL-C was 125 mg/dL, and HDL-C was 40 mg/dL. On 05.12.2024 the blood investigations showed the following lipid levels: TC was 178 mg/dL, TGs were 152 mg/dL, LDL-C was 108 mg/dL, and HDL-C was 42 mg/dL. This information is summarized in Table 2 (see PDF).

## Discussion:

Dyslipidemia can arise from genetic defects in the production of lipoproteins or in the metabolism of lipoproteins; however, in most cases, it results from an unhealthy diet and lifestyle, such as excessive tobacco use and alcohol consumption. It may also be linked to other health issues, including obesity, infections, diabetes, and obstructive liver disease, or it can be caused by certain medications, such as beta blockers and steroids. Available hypolipidemic drugs can lead to various side effects, and it's crucial to understand their impact on health. Statins are generally well tolerated by the majority of patients; however, about 1% may experience a concerning rise in liver enzymes such as alanine aminotransferase (ALT) and aspartate transaminase (AST). While statin therapy is not recommended for those with liver disease, research shows it does not worsen liver function in patients with fatty liver, chronic hepatitis C, or primary biliary cirrhosis. On the other hand, individuals taking crystalline or extended-release niacin frequently experience significant increases in ALT levels; the risk of liver toxicity is particularly high with slow-release niacin. Therefore, it is strongly advised to monitor ALT levels at baseline and again 1 to 3 months after starting statin or niacin treatment. Furthermore, patients using fibrates often see plasma creatinine levels rise by 15-20%, with even greater increases in those who have existing renal issues [10]. Several studies in Asian countries, including Indonesia and China, have explored the development of polyherbal remedies to address dyslipidemia [11]. KM, scientifically known as *Phyllanthus amarus Schum*. & *Thonn* is a distinct single-herb remedy used in Siddha medicine. It is indicated for treating several conditions, including jaundice, liver diseases, diabetes mellitus, urogenital disorders, syphilitic ulcers, and leucorrhea. KM is particularly recognized for its ability to address liver diseases within Siddha practices. KM demonstrates various properties, including hepatoprotective, anti-hepatitis B, antioxidant, antidiabetic, anti-inflammatory, antinociceptive, antimicrobial, antitumor, and antiviral effects [12]. The antihyperlipidemic activities of the aqueous extract from the whole herb of *P. amarus* were analyzed in streptozotocin (STZ)-induced diabetic male Wistar albino rats, showing a significant reduction in lipid levels. Additionally, a study on the hydro-alcoholic extract of the leaves (HAEPA) of *P. amarus* demonstrated its *in vivo* anti-hyperlipidaemic potential using a cholesterol diet-induced hyperlipidemia model in rats, with marked hypolipidemic activity observed at doses of 300 and 500 mg/kg [13]. Moreover, research by Bavara *et al.* indicated that the alcohol extract of Phyllanthus niruri produced a notable lipid-lowering effect on rats [14]. Numerous studies have consistently shown that *P. amarus* possesses significant antihyperlipidemic and hepatoprotective activity. ECM is a siddha sastric preparation and ingredients of ECM are Elettaria cardamomum, Piper nigrum, Syzygium aromaticum, Mesua nagassarium, Taxus buccata, Maranta arunsinace, Zingiber officinale and Saccharum officinarum [15]. Rajalakshmi *et al.* *in vitro* analysis of ECM has antioxidant and anti-inflammatory pharmacological properties [16]. The Elettaria cardamomum is the primary ingredient in ECM. This spice possesses various health benefits, including antihypertensive, anti-atherosclerotic, anti-thrombotic, antioxidant, anti-obesity, lipid-modifying, anti-inflammatory, hepatoprotective, hypocholesterolemic, and antidiabetic properties. A clinical trial conducted by Fatemeh *et al.* demonstrated a notable deduction in TC levels, decreasing from 192.6 to 183.7 mg/dl, as well as a reduction in low-density lipoprotein cholesterol (LDL-C) levels, which fell from 118.1 to 110.5 mg/dl. Additionally, a study by Agashi *et al.* showed a significant decrease in triglyceride (TG) levels, dropping from 158.4 to 125.8 mg/dl. Preclinical studies also indicate that Elettaria cardamomum has potent antihyperlipidemic properties [17].

ABK is a herbometalic Siddha preparation consisting of iron, ferric oxide, lemon, and Eclipta prostrata. Linn. It is indicated for the treatment of anemia, grey hair, and generalized weakness. A study conducted by Elakkiya *et al.* demonstrated that ABK has a potent hepatoprotective effect against paracetamol-induced liver damage in a zebrafish model [18]. The ingredients of ABK also exhibit significant antioxidant and anti-ageing properties [19]. NMK is a polyherbal Siddha formulation used to treat various conditions, including ascites, inflammation, edema, absolute urinary retention or suppression of urine, and complications associated with anemia [20]. The ingredients of NMK have demonstrated hepatoprotective, antihyperlipidemic, diuretic, antiurolithiatic, antimicrobial, anti-inflammatory, antioxidant, immunomodulatory, and nephroprotective properties [21]. The previous literature reviews and preclinical studies showed that the given Siddha medicines exhibited promising hepatoprotective, antihyperlipidemic, antioxidant, and nephroprotective properties. According to Siddha literature, there is no direct term for hyperlipidemia; however, it is classified under liver diseases. The Siddha system recommends specific internal medications for this condition. A fundamental principle in Siddha medicine is that the vitiation of kapam and Pitham humour is a primary cause of hyperlipidemia. Therefore, managing hyperlipidemia in Siddha practice focuses on regulating the vitiated humors. Siddha literature indicates that the tastes of bitter, pungent, astringent and sweet help regulate vitiated kapam and Pitham humour [22]. To address hyperlipidemia, Siddha literature suggests using herbs with these tastes. The therapeutic formulations above contain ingredients that majorly have bitter, pungent, astringent, and sweet properties. It represents Table 3 (see PDF). These properties not only help regulate the vitiated humor but also improve digestion and enhance liver metabolism.The blood lipid investigations showed that the TG levels had moderately decreased, while TC and LDL-C levels showed a mild reduction. Additionally, the HDL-C level increased from 33 mg/dL to 42 mg/dL. Even within the normal range of triglycerides (TGs) at less than 150 mg/dL, there is an increased risk of cardiovascular disease (CVD). Previous research has denoted a crucial interaction between TG levels and CVD risk. In men, the risk seems to rise with increasing TG levels, peaking around 100 mg/dL, while in women, the risk increases up to approximately 200 mg/dL [23]. Our study observed a decrease in TG levels from 301 mg/dL to 152 mg/dL. This result indicates a remarkable reduction in TG levels. Increased cholesterol levels have been linked to a higher risk of cardiovascular disease (CVD), whereas decreased cholesterol levels are associated with a lower risk among young adults. Many previous studies have examined the relationship between cholesterol levels and CVD risk in this age group [24]. In this case report, total cholesterol (TC) was reduced by 178 mg/dL, dropping from 205 mg/dL, while LDL-C decreased by 108 mg/dL, going down from 127 mg/dL. Numerous early epidemiological studies have consistently shown a linear inverse relationship between HDL-C levels and CVD events. Literature reviews and previous research indicate that for every 1 mg/dL increase in HDL-C levels, there is a corresponding 3% to 4% reduction in cardiovascular mortality. This suggests that increasing higher HDL-C levels could lower the risk of CVD events. A study conducted by Yang *et al.* provides compelling evidence of the protective role of high-density lipoprotein cholesterol (HDL-C) in mitigating clinical cardiovascular risk. The researchers analyzed data from a large nationwide cohort comprising 343,687 individuals who underwent routine health examinations. Their findings indicate a strong association between lower HDL-C levels and an increased risk of mortality as well as cardiovascular disease events, including MI and stroke. Notably, extremely high HDL-C levels did not correlate with adverse health outcomes. When examining cardiovascular mortality and the risk of MI, it was observed that the lowest risk occurred at the higher end of HDL-C levels. This finding reinforces the well-established inverse relationship between HDL-C levels and CVD risk. Specifically, elevated HDL-C levels are correlated with a decreased risk of cardiovascular mortality and myocardial infarction [25]. The Siddha therapeutic regimens have shown remarkable hepatoprotective, antihyperlipidemic, and antioxidant effects, significantly enhancing liver metabolism and effectively lowering lipid levels. This case report highlights the promising results achieved through Siddha intervention, underscoring its potential as a valuable approach to improving liver health.

## Conclusion:

In this present comprehensive case report indicates that Siddha medicines exhibit significant efficacy in the management of hyperlipidemia. In conjunction with dietary modifications and lifestyle changes, these treatments have the potential to enhance liver metabolism effectively. Furthermore, it is advisable to pursue additional clinical studies involving a larger cohort of patients to comprehensively evaluate the effectiveness of Siddha management in addressing hyperlipidemia. Such research endeavors could contribute valuable insights to the field and support the development of robust treatment strategies for affected individuals.

## Funding:

None

## Author contributions:

Conceptualization, N. N. J.; methodology, N. N. J. and H. N. V.; software, N. N. J. and H. N. V.; validation, N. N. J. and H. N. V and S. R.; formal analysis, N. N. J. and S. L.; investigation, N. N. J. and H. N. V.; data curation, N. N. J. and H. N. V and S. L.; writing-original draft preparation, N. N. J. and H. N. V and S. R.; writing-review and editing, N. N. J. and H. N. V and S. R.; supervision, S. R. All authors have read and agreed to the published version of the manuscript.

## Ethics approval and consent to participate:

Written permission for publication of this case study was obtained from the patient.

## Availability of Data:

The datasets analyzed in this study can be found within the manuscript.
